# Insulin, insulin-like growth factor–1, insulin receptor, and insulin-like growth factor–1 receptor expression in the chick eye and their regulation with imposed myopic or hyperopic defocus

**Published:** 2011-05-31

**Authors:** Alexandra Marcha Penha, Frank Schaeffel, Marita Feldkaemper

**Affiliations:** University Eye Hospital, Institute for Ophthalmic Research, Section of Neurobiology of the Eye, Tuebingen, Germany

## Abstract

**Purpose:**

Insulin stimulates eye growth in chicks and this effect is greatly enhanced if the retinal image is degraded by the defocus of either sign. However, it is unclear whether the insulin receptor (IR) is expressed at all in the chicken retina in animals 1–2 weeks post-hatching. We have investigated IR expression and whether IR transcript abundance varies in the fundal layers. To elucidate the possible role of insulin and insulin-like growth factor (IGF)-1 signaling in eye growth regulation, mRNA (mRNA) levels were measured for insulin, IGF-1, IR, and IGF-1 receptor (IGF-1R) during imposed negative or positive defocus.

**Methods:**

Chicks were treated binocularly with positive or negative spectacle lenses for 4 or 24 h, or they remained untreated (n=6, for each treatment group). Northern blot analyses were performed to screen for transcription variants in the different fundal layers of untreated animals. Real-time PCR was used to quantify *IR*, *IGF-1R*, *IGF-1*, and insulin mRNA levels in the different fundal layers of the chick eye in the three treatment groups.

**Results:**

*IR* mRNA was found in all the studied tissues, although there is evidence of tissue-specific transcript variations. Three major transcripts were detected for *IR*. The brain, retina, and choroid showed the longest transcript (4.3 kb), which was not present in the liver. Nevertheless, the liver and brain showed a second transcript (2.6 kb) not present in the retina and choroid. A short transcript (1.3 kb) was the predominant form in the liver and choroid, and it seems to be present in the retinal pigment epithelium (RPE) and sclera as well. In the retina, no significant gene expression changes were found when defocus was imposed. Interestingly, in the RPE, both *IR* and *IGF-1R* were already downregulated after short periods (4 h) of positive lens wear. In contrast, *IR* and *IGF-1R* were upregulated in the choroid and fibrous sclera during treatment with negative, but not positive, lenses.

**Conclusions:**

Differences observed in the *IR* transcript length in different tissues suggest possibly different functions. The differential regulation of *IR* and *IGF-1R* in the RPE, choroid, and fibrous sclera is consistent with their involvement in a signaling cascade for emmetropization.

## Introduction

The prevalence of myopia in the human population has dramatically increased in developed regions of Asia [1], but also in Western societies [2] during the last decades. It is estimated that approximately 30% of the worldwide population is currently myopic [3]. Genetic, as well as environmental, factors have been implicated in the development of myopia, but the relative importance of genes versus environment remains controversial [4]. Myopia can be artificially induced in animal models like chicks [5], tree shrews [6], monkeys [7,8], and guinea pigs [9] by placing negative lenses, which induce hyperopic defocus [10], in front of the animal’s eye. The shift of the focal plane behind the photoreceptor layer triggers substantially increased eye growth. Furthermore, the choroid thins. In contrast, positive lenses, imposing myopic defocus, slow the rate of ocular elongation and the choroid thickens by up to a factor of 3 in chicks [11].

Among the retinal transmitters and modulators implicated in eye growth regulation are vasoactive intestinal polypeptide [12,13], dopamine [14–16], retinoic acid [17–19], glucagon [20–22], insulin [23,24], γ-aminobutyric acid [25], and growth factors, such as transforming growth factor and basic fibroblast growth factor [26,27]. In addition, it has been shown that the transcription factor Egr-1 (called ZENK in chicks) may be involved [28–30].

It was previously found that glucagon and insulin have opposite effects on cell proliferation in the retina [31] and on axial eye growth [24,32]. While intravitreal glucagon injections inhibit growth toward myopia in chicks, by slowing axial eye growth rates, insulin not only blocks hyperopia development, which is normally induced by positive lenses, but also induces high amounts of axial myopia that is further increased when negative lenses are worn [32]. In addition, insulin and insulin-like growth factor (IGF)-1 both increase the rate of ocular elongation in eyes not wearing any lenses [24]. Glucagon agonist injections prevent deprivation myopia in a dose-dependent manner [20,33], largely by increasing choroidal thickness [24]. On the contrary, insulin injections cause choroidal thinning in chicks wearing positive lenses, but have no effect on choroidal thickness in animals that have normal vision [32]. When both glucagon and insulin are injected as a cocktail, the growth-promoting effect of insulin is blocked, while the effects of glucagon on choroidal thickness are also suppressed [32]. Interestingly, a very recent study [34] demonstrated a genetic association between IGF-1 and high-grade myopia in an international family cohort. These findings are in line with experimental data from the chicken model of myopia, showing that IGF-1 can promote ocular growth and axial myopia.

So far, only a few studies have targeted IGF-1 and insulin in the eye, apart from those related to their roles in embryogenesis. The human interphotoreceptor matrix displays IGF-1 immunoreactivity, while cultured human retinal pigment epithelium (RPE) cells synthesize and release IGF-1, raising the possibility that the RPE may serve as a source of IGF-1 in vivo [35]. Moreover, cultured embryonic retinal chicken explants contain, synthesize, and release appreciable amounts of IGF-1, which can stimulate the DNA synthesis of retinal explants [36]. Insulin-like immunoreactivity was demonstrated in glial cell culture, but it remains unclear whether this immunoreactivity was due to the binding of circulating pancreatic insulin to insulin receptors (IRs) and/or uptake and storage in these cells, or if insulin is indeed locally synthesized. In situ hybridization studies showed that Müller cells contain mRNA (mRNA) necessary for de novo synthesis of insulin or a closely homologous peptide [37]. Because Müller cells contain glycolytic enzymes and can synthesize and store glycogen [38], it has been suggested that insulin produced in the retina may play a role in glucose or amino acid metabolism. There is evidence that retinal cells are capable of synthesizing preproinsulin mRNA, raising the possibility that insulin is involved in intracellular (autocrine) and intercellular (paracrine) signaling [39]. Moreover, it has been speculated that insulin acts like a growth hormone during development to control retinal differentiation. Later, it may act as a modulator of neurotransmission within the retina [39]. The presence of insulin in the developing retina before pancreatic insulin synthesis is initiated [40] suggests an important role of insulin in the retina, perhaps as a growth or trophic factor. From the rat brain, it is already known that insulin can modulate neurotransmission by increasing the efficiency of neuroactive amino acid reuptake [41]. In addition, insulin has been shown to affect brain monoamine metabolism [42] and dopamine release [43].

The polypeptide hormones insulin and IGF-1 exert their biologic effects by binding to distinct transmembrane receptors on the surface of the target cells. Although the receptors for insulin and IGF-1 are, like their ligands, highly homologous [44,45], they are known to have different, but partially overlapping, physiologic functions [46]. While insulin is known to be a key regulator of physiologic processes such as glucose transport and glycogen and fat biosynthesis [47], IGF-1 is believed to mediate the effects of growth hormone and play a role as a paracrine growth factor [48]. The levels of the IR are regulated during development, and it is likely that changing the receptor level while keeping the level of insulin constant may be a regulatory mechanism [49]. Analysis of the protein structure has revealed that receptors for IGF-1 and insulin belong to a family of cell surface glycoproteins that share a cytoplasmic tyrosine-kinase function [50,51]. Both are oligomers composed of two types of subunits: α-subunits containing the hormone-binding site, and β-subunits, which are phosphorylated after binding of the ligand. The alpha and beta subunits are encoded by a single gene. Ligand interaction with the extracellular portions of these receptors activates intracellular tyrosine-kinase activity, and generates a biologic signal that is thought to be specified by structural determinants in the cytoplasmic domain. The presence of IR/IGF receptor hybrids was demonstrated in proliferative neuroretina. These receptors were considered to be physiologically relevant for the action of the locally produced proinsulin found in early neurogenesis [52]. Two types of IGF receptors on nerve cell membranes from the murine and human central nervous system (CNS) were identified based on their binding specificity, subunit structure, kinase activity, and interaction with antibodies to insulin receptor [53]. In the CNS, insulin receptors are also composed of two types of subunit, but the size of the α-subunit is significantly smaller, whereas the β-subunit is similar to that of other cell types [54–56]. The differences in the composition of IR in neuronal and nonneuronal cells suggest a unique function for IR in neural networks [57].

Because of these fundamental differences between IR molecules in the brain and peripheral target tissues [54], the first objective of this study was to investigate which transcript variants of IR exist in the fundal layers of the eye, compared to the liver and brain, to learn more about which variants might be involved in eye-growth regulation. The second objective was to study changes in mRNA levels for insulin, *IGF-1*, *IR*, and IGF-1 receptor (*IGF-1R*), after defocus was imposed in the retinal image for 4 or 24 h, a condition that is known to induce axial refractive errors.

## Methods

### Treatment of animals

Ten-day-old male White Leghorn chickens were raised under a 12 h:12 h light-dark cycle, and treated binocularly either with plus (+7D) or minus (−7D) lenses for 4 (n=6) and 24 h (n=9), respectively. In addition, a separate control group was used for each treatment duration. To attach the lenses, Velcro rings were glued onto the feathers around the eyes a few hours before the lens treatment was started. The experimental treatment was in accordance with the ARVO Statement for Care and Use of Animals in Ophthalmic and Vision Research and was approved by the university commission for animal welfare.

### Tissue preparation

The chicks were sacrificed by an overdose of diethyl ether between 1 and 3 PM Eyes were enucleated and vertically cut with a razor blade, discarding the anterior part containing the lens. The vitreous body was removed and the pecten was cut out. From the posterior part of the eye, a biopsy punch of 8 mm was made and placed in a Petri dish that was filled with ice-chilled saline. The different fundal layers were carefully separated under visual control of a dissecting microscope. In addition, forebrain and liver samples were dissected from four untreated animals. All the tissues were immediately collected in RNAlater (Qiagen, Hilden, Germany), immediately frozen in liquid nitrogen, and stored at −80 °C until RNA extraction. In general, the right eyes were taken for further analysis. Only when the separation of the fundal layers was not optimal was the left eye studied instead.

### Total RNA extraction and cDNA synthesis

Different RNA extraction methods were used for northern blot and real-time PCR analyses. For northern blot analysis, total RNA from the liver, brain, retina, RPE, choroid, and sclera was isolated using TRIzol (Invitrogen, Karlsruhe, Germany) according to the manufacturer’s instructions. For real-time PCR analyses, the RNeasy Mini kit (RNeasy Mini Kit; Qiagen, Hilden, Germany) was used following the manufacturer’s instructions. All tissues were homogenized in the respective lysis buffer for 1 min, at a range of speed that increased in four steps from 11,000 to 20,000 rpm (Diax 900 Homogenizer; Heidolph, Ketheim, Germany). All RNA samples were treated with DNase I (DNA-*free* Ambion, Darmstadt, Germany) and the respective yield was measured by spectrophotometry at 260 and 280 nm. The optical density (OD_260_/OD_280_) ratios were calculated to ensure the quality of the isolated RNA, and samples with a ratio between 1.8 and 2.0 were used for further analysis. The integrity of the RNA samples was confirmed by agarose-gel electrophoresis. Thereafter, 1 µg of brain, liver, retina, RPE, and choroid, and 0.5 µg of both sclera layers were reversed transcribed by Moloney Murine Leukemia Virus (M-MLV) reverse transcriptase (Promega, Mannheim, Germany) using 0.25 µg oligo(dT)_15_ and 0.025 µg random hexamer primers (Invitrogen) in a final volume of 15 µl.

### Semiquantitative real-time polymerase chain reaction

Table 1 shows all of the specific primer sequences used for semiquantitative real-time PCR, the respective amplicon size, and the NCBI accession number. Primer design was performed using the web-based program Oligo Explorer 1.4 (Gene Link, Hawthorne, NY). The specificity of the PCR reactions was verified by melting-curve analysis and agarose-gel electrophoresis, and the PCR products were sequenced to verify their identity. The PCR reactions were performed in a thermocycler (iCycler iQ Real-Time PCR System; Bio-Rad, Hercules, CA) using a fluorescence detection kit (QuantiTect SYBR Green PCR kit, Qiagen). Primer annealing was executed at 59 °C for 30 s and elongation at 72 °C for 20 s. Every single reaction, with a final volume of 15 μl, contained a primer concentration of 0.6 μM, a template amount corresponding to 2 ng of RNA, and the master mix of the fluorescence kit. Each sample was analyzed in triplicate and the fluorescence signal was measured with every cycle at 72 °C. In addition, but only for insulin, a hydrolysis probe and primers designed by Biomers (Ulm, Germany) were used to verify the specificity of insulin mRNA expression (Table 1). To compare the amplification of different regions from the *IR* mRNA sequence, different pairs of primers comprising different exons were designed based on the sequence provided by the Entrez and Ensembl databases (Table 2).

**Table 1:** Sequences of the specific primers used for real time-PCR amplification

**Gene**	**Forward primer (5′-3′)**	**Reverse primer (5′-3′)**	**Amplicon size**	**NCBI accession**
β-actin	CTGAACCCCAAAGCCAAC	CACCATCACCAGAGTCCATCAC	147 bp	NM_205518
HPRT	TGGCGATGATGAACAAGGT	GCTACAATGTGGTGTCCTCCC	162 bp	NM_204848
Insulin	CTTCTGGCTCTCCTTGTCTTTT	CAAGGGACTGCTCACTAGGGGC	172 bp	NM_205222.2
Insulin Receptor	CGCTGAGAATAACCCTGGTC	GCTGCCATCTGGATCATTTC	60 bp	XM_001233398.1
IGF-1	CTTCAGTTCGTATGTGGAGACA	GATTTAGGTGGCTTTATTGGAG	167 bp	NM_001004384.1
IGF-1 Receptor	TCCAACACAACACTGAAGAATC	ACCATATTCCAGCTATTGGAGC	167 bp	NM_205032.1
Insulin (hydrolysis probe primers)	GGCTCTCTACCTGGTGTGTG	CTCGCTTGACTTTCTCGTATTCC	149 bp	NM_205222.2
Insulin-hydrolysis-probe	CACTCCTGCCTCGCCACGC			

IR – insulin receptor; IGF-1 – insulin-like growth factor-1; IGF-1R – IGF-1 receptor

**Table 2:** Primer sequences used to compare the amplification of different regions of the insulin receptor mRNA

**Gene**	**Region on the sequence**	**Primer (5′-3′)**	**Amplicon size**
IR-LD	L2-(binding) domain	GGTCGTATGCCTTGGTTTC	118 bp
		AGCTGGCGAAGATTCTGG	
IR-TK	Tyrosine kinase domain	CGTCCACCACCAACACTG	58 bp
		TGCCATCAGCGATCTCTG	
IR-TK2	Tyrosine kinase domain	GTTCACAGAGACCTGGCA	103 bp

### Northern blot analysis

Differences in transcript size were analyzed by northern blotting. Biotin-labeled antisense probes were designed using Oligo Explorer 1.4 based on the published mRNA chicken sequences in the Entrez and Ensembl databases. Two specific probes for IR were used for northern blot analysis (Table 3). Approximately 1 μg of RNA was run in a 0.8% formaldehyde-agarose gel, blotted overnight onto a positively charged nylon membrane (Roche, Mannheim, Germany), and crosslinked upon exposure to ultraviolet light. Blots were hybridized overnight with 100 ng/ml of biotin-labeled IR probe at 50 °C. The next day, the membranes were washed twice for 5 min each with 2× Saline-Sodium-Citrate buffer (SSC)/0.1% sodium dodecyl sulfate (SDS) at 42 °C, followed by two additional washes, for 15 min each, with 0.5× SSC/0.1% SDS, at the same temperature (1× SSC buffer contains 0.15 M NaCl and 15 mM Na_3_-citrate*2 H_2_O, pH 7.0). Chemiluminescence detection was performed with the Chemiluminescent Nucleic Acid Detection Mode kit (Thermo Scientific GmbH, Ulm, Germany). Blots were exposed to X-ray films Curix HT1 (AGFA, Leverkusen, Germany) and the time of exposure was adjusted as needed to obtain the desired signal strength. Liver RNA was used as a positive control for *IR* expression, and the brain was used as a nervous tissue control. Two to four samples per tissue were used for northern blot analysis with probe 1 (Table 4), but only one retina, brain, and liver sample was used for northern blot analysis with probe 2.

**Table 3:** Sequences of the specific probes used for northern blot analysis.

**Gene**	**Probe**	**Probe sequence**	**Region**
Insulin receptor	1	AGCCATCTGGATCATTTCTCTCAGTGTTGGTGGTGGACG	Tyrosine kinase domain
	2	TTCCTCCACGGATATTTATAACCAAGCTCCCATTAACAACTGTGCAGCCA	L2-binding domain

**Table 4:** Summary of treatment groups.

**Title of the experiment**	**Lens treatment**	**Duration of treatment**	**Tissues**	**n**
Northern blot analysis of IR expression	Without lenses^1^	-	Liver	4
		-	Brain	4
		-	Retina	4
		-	RPE	2
		-	Choroid	4
		-	Sclera (both layers)	2
Comparison of amplification of different regions of IR	Without lenses^1^	-	Liver	4
		-	Brain	4
		-	Retina	4
		-	RPE	4
		-	Choroid	4
		-	Fibrous sclera	4
		-	Cartilaginous sclera	4
Insulin, IGF-1, IR and IGF-1R mRNA expression in the ocular fundal layers	Without lenses	4 h	Retina	6
	Plus lenses	4 h	RPE	4
	Minus lenses	4 h	Choroid	4
	Without lenses^1^	24 h	Retina	9
	Plus lenses	24 h	RPE^2^	9
	Minus lenses	24 h	Choroid	9
		24 h	Fibrous sclera	9
		24 h	Cartilaginous sclera	9

^1^Same animals. ^2^The RPE tissues from both eyes were analyzed separately for all 24 h treatment groups. We then calculated the mean of both eyes before calculating the grand mean (See section “Tissue preparation”).

### Statistics and data analysis

Statistical analysis was done based on the quantification cycle (C*q*) values of the PCR products. To test the primers’ efficiency, a dilution curve was created using template amounts ranging from 0.5 to 16.0 ng per well. The efficiency (*E*) for each primer was calculated according to the formula: *E*=10^(−1/slope)^, giving a value between 1 and 2, whereby 1 corresponds to 0% efficiency and 2 to 100%. The slope (*m*) was determined by plotting the mean of C*q* of each of the cDNA dilution samples versus the logarithm of the sample concentration. The efficiencies were 2.03 for β-actin, 2.11 for hypoxanthin-guanin-phosphoribosyl-transferase (*HPRT*) 2.02 for *IR*, 1.97 for *IGF-1*, and 1.98 for *IGF-1R*. The mean normalized expression (MNE) [58] was used to compare relative expression levels among different groups and was calculated according to the following formula, where *E* is the primer efficiency, reference corresponds to β-actin, and the targets are *IR*, *IGF-1*, and *IGF-1R*:

$$MNE=\frac{{\left(E_{\text{reference}}\right)}^{Cq_{\text{reference, }mean}}}{{\left(E_{\text{target}}\right)}^{Cq_{\text{target},mean}}}$$

MNE values were first analyzed using an outlier calculator (GraphPad, La Jolla, CA). Then, one-way ANOVA (ANOVA) was applied for statistical comparison between the different treatment groups. A significant ANOVA (p<0.05) was followed by a Student's *t*-test for post hoc analysis. Statistical tests were performed using JMP version 7 software (SAS Institute, Cary, NC).

## Results

### Northern blot analysis of insulin receptor expression in untreated tissues

**Figure 1:**
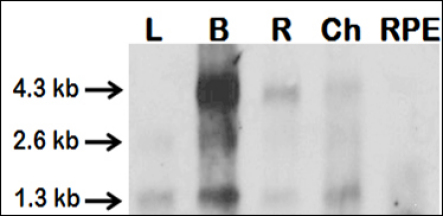
Northern blot showing the expression pattern of the insulin receptor mRNA (probe 1) in the liver (L), brain (B), and different fundal ocular layers: the retina (R), choroid (Ch), and retinal pigment epithelium (RPE). Three major transcripts with 4.3, 2.6, and 1.3 kb were found, although the pattern was different among the studied tissues.

Northern blots were used to compare the transcript length of the *IR* (Figure 1) in neuronal and nonneuronal tissues. The emphasis in the northern blot analyses was placed on the investigation of transcript variants among different tissues and not on the quantification of the *IR* mRNA levels in those tissues. Therefore, a loading control was not used. Figure 1 shows a northern blot result for *IR* expression in the liver, brain, retina, choroid, and RPE.

With probe 1, the brain and the retina showed two transcripts, approximately 4.3 kb and 1.3 kb long, with the longer transcript being more abundant. In addition, the brain showed a third band of about 2.6 kb. This transcript was also found in the liver, together with the more abundant 1.3 kb transcript and a small transcript of 0.4 kb, which might be a degradation product (not shown). The choroid, like the brain and retina, also expressed two transcripts of 4.3 and 1.3 kb, but in this tissue, the shorter transcript was more prominent than the longer one. The RPE contained only very small amounts of *IR* mRNA, and mainly the shorter variant. In addition, we analyzed two scleral samples (combined fibrous and cartilaginous layer). They expressed the 1.3 kb transcript and two smaller transcripts of approximately 0.8 kb and 0.4 kb (results not shown). With probe 2, which was complementary to a part of the L2-binding domain, the retina and the brain only showed one very strong band, corresponding to 5.3 kb. The same band was found in the liver, although it was very faint, in combination with a 2.0 kb band.

### Comparison of amplification of different regions of the insulin receptor mRNA sequence, by real-time PCR

**Figure 2:**
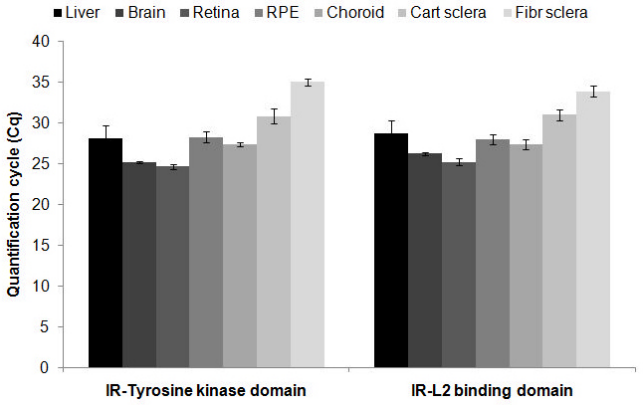
Quantification cycle values for two different regions of the insulin receptor sequence in different tissues are shown. All tissues expressed the insulin receptor tyrosine kinase domain mRNA as well as the insulin receptor L2-rich binding domain mRNA. The sample size is 4 animals per tissues. Error bars represent the standard error of the mean.

Three pairs of primers were designed to amplify different parts of the *IR* sequence. The first primer pair, called insulin receptor ligand-binding domain (IR-LD), amplified a part of the sequence corresponding to the L2 domain in the receptor protein. The leucine-rich L2 domain is involved in the ligand binding and is encoded by exon numbers 4 and 5. The second primer pair, IR-tyrosine kinase (TK), was designed to amplify a fragment that after translation belongs to the tyrosine-kinase domain, which is a catalytic domain with phosphotransferase activity, and comprises exons 16 and 17. The mentioned exons are localized on the longest transcript sequence for the *IR* mRNA based on the Ensembl database. Based on the results (Figure 2), all tissues expressed mRNA for the tyrosine-kinase domain and the L2 (binding domain), although in different amounts. For both the IR-TK and IR-LD, the retina and brain showed the highest amounts, followed by the choroid, RPE, liver, and cartilaginous and fibrous sclera. The third primer pair also amplified a part of the tyrosine-kinase domain and comprised exons 17 and 18. Concerning the IR-TK2 region, no specific PCR product was obtained in most tissues; only the retina and liver showed a very low expression (data not shown).

### Insulin receptor, IGF-1 receptor, *IGF-1*, and insulin mRNA expression in ocular fundal layers of untreated animals

**Figure 3:**
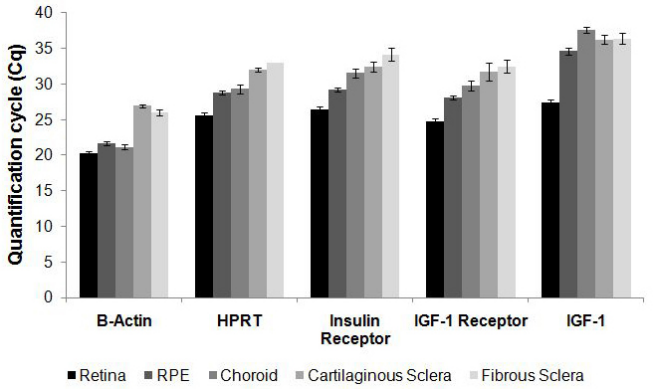
Quantification cycle values for all genes in all fundal layers are shown. The mRNA for the insulin receptor and insulin-like growth factor receptors is most abundant in the retina, followed by the RPE, choroid, cartilaginous and fibrous sclera. The sample size is 6 animals per tissues. Error bars represent the standard error of the mean.

The expression levels of *IR*, *IGF-1R*, *IGF-1*, and insulin were measured and compared in all fundal layers of untreated chicks. The results are shown in Figure 3, with a higher quantification cycle threshold corresponding to a lower amount of mRNA. Both receptors and *IGF-1* were detected in all tissues, but besides the retina, the amount of *IGF-1* mRNA was very low. In addition, insulin mRNA was detected in the retina, but at very low concentrations, and in the choroid with even lower levels than in the retina. Retinal insulin expression was confirmed when an insulin-specific hydrolysis probe was used. The usage of a hydrolysis probe offers a high specificity, because hybridization and fluorescence will only occur if the target DNA sequence exactly matches the hydrolysis probe sequence (for further information, see reference [59]). The results for insulin mRNA quantification are not shown in detail, since the expression level was too low to be precisely quantified.

Comparing the mRNA amount between different fundal layers, it turned out that the mRNA for both receptors was most abundant in the retina, followed by the RPE, choroid, and cartilaginous and fibrous sclera. In the fibrous sclera, the C*q* values for all the genes were higher, and therefore, mRNA levels were lower for the reference genes (β-actin and *HPRT*), as well as for all the other genes.

### Insulin mRNA expression in the retina, retinal pigment epithelium, and choroid after lens treatment

Although only very low amounts of insulin mRNA were detectable in the retina and choroid of untreated animals, lens treatment might upregulate this amount. The expression level of insulin was therefore measured and compared in the retina, RPE, and choroid after 4 and 24 h of lens treatment. However, no significant increase in insulin mRNA levels was detected under any of these conditions.

### Insulin receptor, IGF-1 receptor, and *IGF-1* mRNA levels in the retina after lens treatment

**Figure 4:**
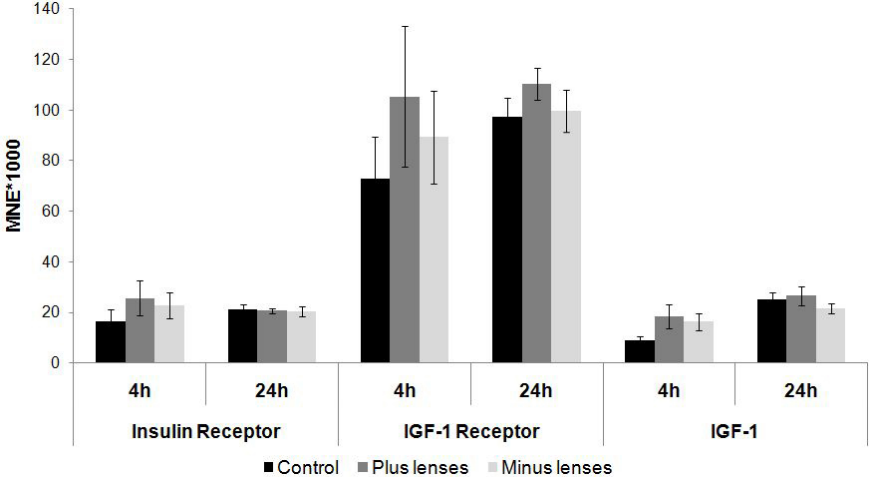
Retinal insulin receptor, insulin growth factor–1 receptor, and insulin-like growth factor-1 mRNA levels after 4 and 24 h of plus (+7D) and minus (−7D) lens treatment. Results are expressed as the mean normalized expression (MNE)±SEM. For the 4 h experiment, six animals per groups were used; nine per group were used for the 24 h experiment. Insulin receptor (IR), insulin-like growth factor (IGF)-1 receptor (IGF-1R), and IGF-1 mRNA levels were not significantly influenced by lens wear in the retina.

Treatment with negative and positive lenses did not significantly alter *IR* or *IGF-1R* mRNA expression levels after 4 or 24 h of lens treatment compared with the appropriate control group (Figure 4). In addition, neither 4 h nor 24 h of lens treatment had a significant influence on *IGF-1* mRNA expression levels.

### Insulin receptor, IGF-1 receptor, and *IGF-1* mRNA levels in the retinal pigment epithelium after lens treatment

**Figure 5:**
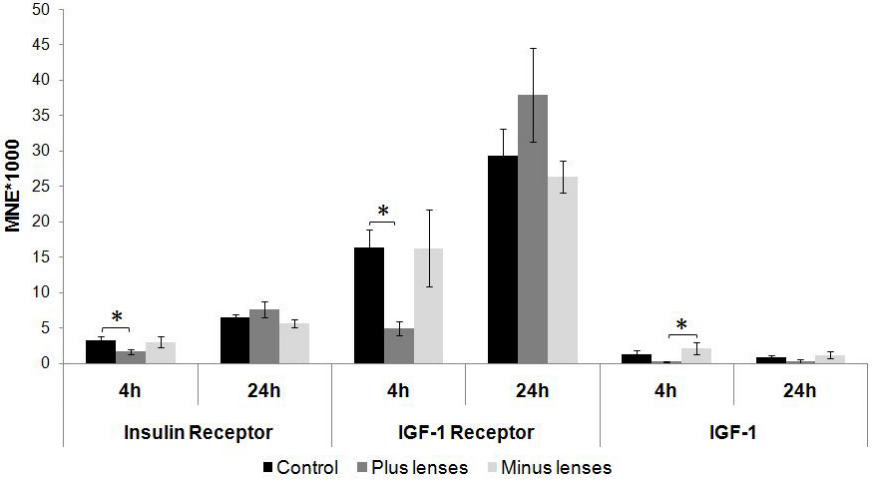
Insulin receptor, insulin-like growth factor-1 and insulin-like growth factor–1 receptor mRNA levels after 4 and 24 h of plus (+7D) and minus (−7D) lens treatment in the retinal pigment epithelium. Results are expressed as the mean normalized expression (MNE)±SEM. For the 4 h experiment, six animals per groups were used; 9 per group were used for the 24 h experiment. Statistically significant differences between the treated groups and the control as determined by one-way ANOVA (ANOVA) are denoted in the graph (* for p<0.05). Insulin receptor (IR) and insulin-like growth factor (IGF)-1 receptor (IGF-1R) mRNA levels were lower after 4 h of plus lens treatment compared to untreated control animals. In addition, IGF-1 mRNA levels were significantly lower after 4 h of plus lens treatment compared to the minus lens–treated animals. After 24 h of lens treatment, there were no significant differences in all genes, between all groups.

Four hours of myopic defocus induced a twofold downregulation of IR and an approximately fourfold downregulation of *IGF-1R* mRNA levels, compared to the respective control groups (Figure 5, ANOVA, *IR*, plus lens versus control p=0.03; *IGF-1R*, plus lens versus control p=0.03). This effect disappeared when the animals were treated with lenses for 24 h. In comparison to the levels in control animals, lens treatment did not significantly influence *IGF-1* mRNA levels after 4 or 24 h. Nevertheless, *IGF-1* mRNA levels were significantly lower after 4 h of positive lens wear compared to 4 h of negative lens wear (ANOVA, minus versus plus p=0.05).

### Insulin receptor, IGF-1 receptor, and *IGF-1* mRNA levels in the choroid after lens treatment

**Figure 6:**
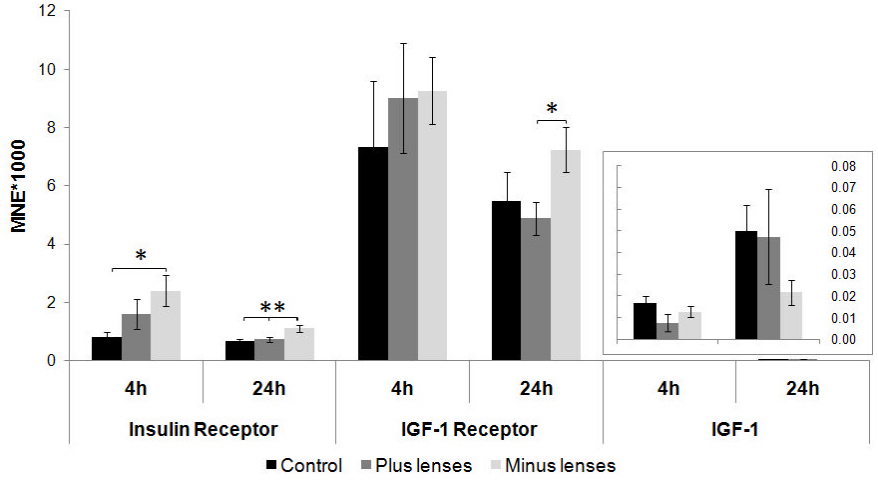
Insulin receptor, insulin-like growth factor–1 receptor, and insulin-like growth factor–1 mRNA levels after 4 and 24 h of plus (+7D) and minus (−7D) lens treatment in the choroid. Results are expressed as the mean normalized expression (MNE)±SEM. For the 4 h experiment, six animals per groups were used; nine per group were used for the 24 h experiment. Statistically significant differences between the treated groups and the control, as determined by one-way ANOVA (ANOVA) are denoted in the graph (* for p<0.05 and ** for p<0.01). mRNA levels for insulin receptor (IR) were significantly increased after 4 h and 24 h of minus lens treatment, and the insulin-like growth factor (IGF)-1 receptor (IGF-1R) mRNA level was higher in the minus lens–treated group compared to the plus lens–treated group after 24 h of lens wear.

In the choroid, treatment with negative lenses for 4 h resulted in an initial threefold increase of *IR* mRNA concentration compared to the control group (Figure 6, ANOVA, minus lens versus control p=0.03). These changes in the minus lens–treated group remained after 24 h, compared with both the control and plus groups (ANOVA, minus lens versus control p=0.004; minus lens versus plus lens p=0.01; Figure 6). *IGF-1R* was also increased in the minus lens–treated group compared to the plus lens group, but only after 24 h of lens treatment. This effect was not as strong as for *IR* (ANOVA, minus lens versus plus lens p=0.05). *IGF-1* mRNA expression in the choroid was very low and difficult to quantify. Within this limitation, no significant changes in *IGF-1* mRNA expression were detected.

### Insulin receptor, IGF-1 receptor, and *IGF-1* mRNA levels in both scleral layers after lens treatment

**Figure 7:**
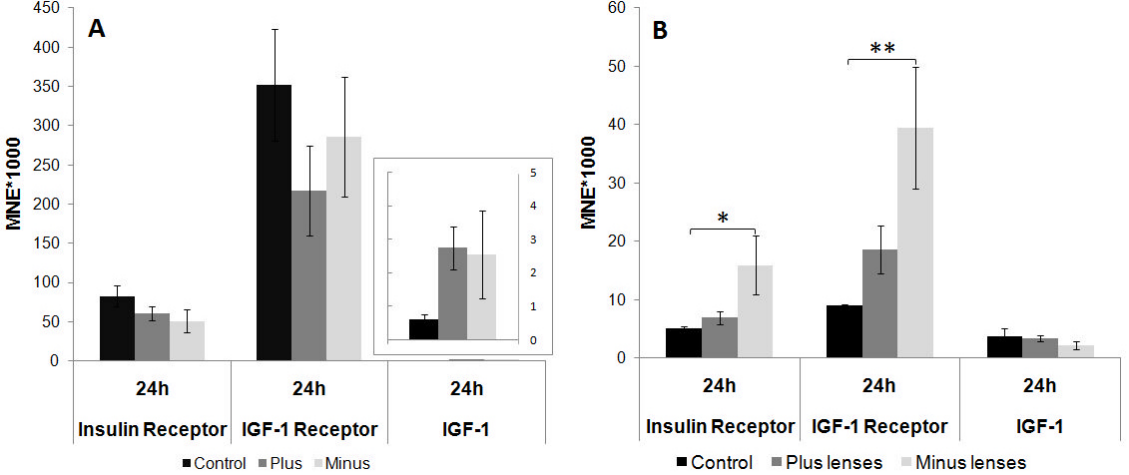
Insulin receptor, insulin-like growth factor-1 receptor, and insulin-like growth factor–1 mRNA levels after 24 h of plus (+7D) and minus (−7D) lens treatment in the cartilaginous sclera (**A**) and fibrous sclera (**B**). Results are expressed as the mean normalized expression (MNE)±SEM. Nine animals were analyzed per group. Statistically significant differences between the treated groups and the control, as determined by one-way ANOVA (ANOVA) are denoted in the graph (* for p<0.05 and ** for p<0.01). In the cartilaginous sclera, the mRNA contents of the two receptors were not significantly different. In the fibrous sclera, the expression of the insulin and the insulin-like growth factor (IGF)-1 receptor (IGF-1R) was higher in the minus lens–treated group compared to controls.

In the cartilaginous sclera, lens treatment influenced neither *IR* nor *IGF-1R* mRNA levels after 24 h of lens treatment (Figure 7A). However, in the fibrous sclera (Figure 7B), 24 h of positive-imposed defocus induced a threefold upregulation of *IR* mRNA levels compared with the control group, and a fourfold upregulation for *IGF-1R* at the same time point (ANOVA, *IR*, minus lens versus control p=0.038; *IGF-1R*, minus lens versus control p=0.005). *IGF-1* was expressed at low levels, especially in the cartilaginous sclera. Lens treatment did not induce a significant change in *IGF-1* mRNA levels in either layer.

## Discussion

### Insulin receptor transcript variants

Significant differences in the transcript sizes of *IR* have been previously described in different tissues and animal species. Four different *IR* transcript variants were reported in chicks (Ensembl database) with transcript lengths varying between 198 and 3,220 bp. All of these can be translated to a protein product, but in the case of the small transcripts, the functions are unknown. In addition, several proteomic studies showed differences in the molecular weight of IR alpha and beta subunits among different tissues [60–63]. IR therefore seems to undergo different transcriptional and translational regulation and posttranslational modifications, including glycosylation or proteolytic cleavage in the CNS [64]. In the brain, the α-subunit of the IR has a lower molecular size compared to that of other tissues. It was therefore hypothesize that insulin exerts its proposed neuromodulatory effects mediated by the specific IRs in the brain [63]. In our study, we found 4.3 kb, 2.6 kb, and 1.3 kb long *IR* transcripts when we used a probe that corresponds to part of the sequence for the IR tyrosine-kinase domain. An mRNA of 4.3 kb can account for a protein as large as IR (1,332 amino acids), whereas the smallest transcript can only encode for parts of the protein. We were able to show that different tissues expressed transcripts of different lengths; the RPE and sclera seem to express mainly short *IR* transcripts. Moreover, the smaller mRNA transcript was the predominant form in the choroid and liver, while the longer transcript was most abundant in the brain and retina. This result may suggest that IRs in the retina and brain have a different function than in the nonneuronal tissues. As expected from the Ensembl database, only one long transcript was detected in the brain and retina when an L2-binding domain–specific probe was used.

One recent publication stated that the chicken retina does not express *IR* mRNA [65]. In contrast, we found relatively high amounts in the retina of our chicks. Since different parts of *IR* were amplified in both studies, we used three different primer pairs for the amplification of different parts of *IR* in an attempt to solve the discrepancy. We found that the mRNA for the insulin-binding domain and the tyrosine-kinase domain are present at moderate levels in the retina. Nevertheless, one PCR product that was designed to amplify a short sequence between exons 17 and 18 could only be detected in a very small amount in the retina and liver, suggesting that this part of the sequence is not efficiently transcribed or differs from that in the literature.

### Possible sites of action for insulin and IGF-1 in the eye

Several lines of evidence support a role of insulin and/or IGF-1 in the control of eye growth [24,32,34], including one strong clue coming from chicken studies, in which it was shown that intravitreal injections of both peptides lead to the development of myopia. The current study aimed to quantify the mRNA expression of both the receptors and their ligands in all fundal layers to gain a broader insight into their role. *IR* and *IGF-1R* were expressed in all tissues, being more abundant in the retina, followed by the RPE, choroid, and cartilaginous and fibrous sclera. Therefore, assuming that the mRNA is translated into protein, all of these are possibly target sites for insulin and IGF-1 action. *IGF-1* mRNA expression was only relatively high in the retina, meaning that only here could a significant amount of IGF-1 be produced. This result corresponds with an older study [66] in which IGF-1-specific transcripts were higher in the neural retina than in the sclera plus choroid plus RPE. Although insulin mRNA expression was detected in the retina and choroid, as confirmed with a specific hydrolysis probe and gel electrophoresis, its level was very low. It was therefore impossible to quantify these low amounts reliably. Lens treatment did not increase insulin mRNA levels in the retina, RPE, or choroid. Taken together, it seems unlikely that changes in the amount of insulin produced by the retina itself influences eye growth. Rather, it is more likely that IGF-1 plays a physiologic role in the retina. Insulin and *IGF-1* mRNA levels in the retina seem to be developmentally regulated, as shown by binding assays, decreasing by about 50% between the embryonic and the post-hatching stages [67]. In the rat retina, it has already been shown that Müller cells may contain the mRNA necessary for de novo synthesis of insulin or a closely homologous peptide [37]. However, the source of insulin in the avascular chicken retina still remains unclear, and due to the very low amounts, it will be difficult to uncover its function.

### Influence of lens treatment on insulin receptor, IGF-1 receptor, and *IGF-1* expression in the fundal layers of the eye

Lens wear influenced *IR*, *IGF-1R*, and *IGF-1* mRNA expression in different fundal layers of the chicken eye, with most changes seen in the RPE, choroid, and fibrous sclera. In our study, the whole retina was used to measure changes in gene expression after induced positive and negative defocus. Insulin and *IGF-1R* mRNA were both highly expressed in the retina, but neither their expression level nor the *IGF-1* mRNA levels were influenced by defocus of 4 and 24 h. In contrast, short plus lens–treatment periods (4 h) led to a strong downregulation of both receptors in the RPE; in addition, the *IGF-1* mRNA levels were much lower in the plus lens group compared to the minus lens–treated animals. Insulin and IGF-1R signaling may therefore be involved in the onset of growth arrest after negative defocus. It is not surprising that the gene expression changes did not persist after 24 h of treatment, since it is known from microarray studies that only a minority of gene expression changes seem to be common to multiple treatment times [68]. This can be interpreted in terms of different mechanisms, one for the onset of increased (minus lens) or decreased (plus lens) eye growth, and the other for maintaining its persistence. An upregulation of *IGF-1R* mRNA expression in the RPE of chicks that were treated with minus lenses for 2 days was recently reported using microarrays [69]. We did not measure the upregulation of this receptor after one day, but as already discussed, time often matters and may explain the different results.

In the present study, we were able to demonstrate that short treatment with plus lenses mainly affected mRNA levels in the RPE, whereas longer and minus lens treatment influenced gene expression level in the choroid and fibrous sclera. The choroid is a thin layer of vascular pigmented tissue with two main physiologic functions: the nourishment of the external retina and the regulation of ocular temperature. Both insulin and *IGF-1* mRNA levels were present at low levels in the choroid, as confirmed with a specific hydrolysis probe and gel electrophoresis, but it was impossible to quantify the low amounts reliably. Nevertheless, the respective receptors levels changed during the treatment. Higher mRNA levels of *IR* were already measured after 4 h, and insulin and *IGF-1R* showed higher expression levels than controls and/or plus lens–treated animals after 24 h of lens wear. Zhu and Wallman [24] recently hypothesized that although it is unknown whether glucagon and insulin first act at the retina, RPE, or choroid, they finally act to change the physiologic state of the choroid, which in turn modulates both choroidal thickness and scleral growth, the latter being manifested as a change in the rate of ocular elongation. Our results support this hypothesis. Especially after minus lens treatment, the only changes in receptor gene expression were detected in the choroid and sclera. Since only low insulin and *IGF-1* mRNA levels were detected in the choroid, is seems unlikely that they are synthesized in significant amounts in this tissue. Instead, the tissue could be a potential target for these growth factors’ action, given that insulin and IGF-1 injections in chicken eyes were shown to induce choroidal thickness changes under some experimental conditions. Insulin increases ocular elongation without thinning the choroid in animals not wearing lenses. Only when plus lenses were attached, which normally cause choroidal thickness, does insulin thin the choroid, as well as accelerating ocular elongation. In contrast, IGF-1 injections increase ocular elongation, together with thickening rather than thinning the choroid [24,32].

In contrast to that of mammals, the sclera of chicks is composed of two layers: an inner cartilaginous layer, containing collagen types II and IV and aggrecan as the predominant proteoglycans, and an outer fibrous layer (like that in mammals), which contains collagen type I and small proteoglycans such as decorin [70]. When the rate of elongation of the eye is visually manipulated, both scleral layers show opposite modulation [62], with the fibrous sclera getting thinner and the cartilaginous becoming thicker during induced eye growth. Interestingly, we were able to show that the fibrous sclera showed a similar upregulation of *IR* mRNA expression as the choroid. One of the reasons for this upregulation of the *IGF-1R* mRNA expression in the fibrous sclera might be that IGF-1 exerts an effect on the developing ocular tissue by influencing the synthesis and degradation of the extracellular matrix in chicks [71]. In the guinea pig model, it was already shown that IGF-1 can induce fibroblast proliferation in a dose-dependent manner through the signal transducer and activator of transcription 3 (STAT3) signaling transduction pathway [72,73]. No lens-induced changes in gene expression were detected in the cartilaginous sclera. Compared to the fibrous sclera, the cartilaginous sclera had higher mRNA levels for all measured genes. This result is consistent with a previous study by Schippert et al. [74]. These authors also showed that the fibrous sclera generally has lower mRNA levels than the cartilaginous sclera in untreated chicks. Co-cultures already demonstrated that the choroid can influence the underlying sclera, for example by changing proteoglycan synthesis in the sclera [75]. Retinoic acid, the synthesis of which is influenced in opposite directions by positive and negative defocus in both the retina and choroid, has been shown to affect proteoglycan synthesis in the chick sclera [17]. Moreover, retinoic acid might interact with the IGF-1 signaling by changing the level of IGF-binding proteins and thereby modulating scleral IGF-1 levels [76].

### Comparison to studies in humans

Recent epidemiological and retrospective case series studies in humans underlined a role of IGF-1 as regulator of ocular growth, at least in patients with primary growth hormone insensitivity [77], children with growth hormone deficiency [78], and children born preterm [79]. Low IGF-1 serum concentrations were associated with hyperopia in these studies. These results are consistent with our animal study showing an association of reduced *IGF-1* mRNA levels with the development of hyperopia in the RPE of chicks. Interestingly, patients with primary growth hormone insensitivity who received IGF-1 therapy showed a tendency toward mild myopia. These findings point toward a role of IGF-1 as a growth signal in humans as well as in chicks.

### Implications and summary

In summary, we found that a short exposure to myopic defocus (plus lenses) leads to a downregulation of insulin receptor and *IGF-1R* receptor expression in the RPE. In contrast, hyperopic defocus, imposed by minus lenses (but not myopic defocus) significantly increased their expression levels in the choroid. Similar changes were seen in the fibrous sclera. Taken together, the current study supports a role of insulin and/or IGF-1 signaling during eye growth. Whether different *IR* transcript variants found in the retina and choroid are also translated into proteins with different functions needs to be shown in the future.
